# Pedigree and genotype errors in the Framingham Heart Study

**DOI:** 10.1186/1471-2156-4-S1-S41

**Published:** 2003-12-31

**Authors:** Gerry Brush, Laura Almasy

**Affiliations:** 1 Department of Genetics, Southwest Foundation for Biomedical Research, San Antonio, Texas, USA

## Abstract

The pedigree and genotype data from the Framingham Heart Study were examined for errors. Errors in 21 of 329 pedigrees were detected with the program PREST, and of these the errors in 16 pedigrees were resolved. Genotyping errors were then detected with SIMWALK2. Five Mendelian errors were found following the pedigree corrections. Double-recombinant errors were more common, with 142 being detected at mistyping probabilities of 0.25 or greater.

## Background

Because linkage analysis observes the co-segregation of marker alleles and phenotype, there is a concern that errors in pedigrees or genotypes could result in false-negative or false-positive results. The use of Genetic Analysis Workshop (GAW) data to examine the presence and nature of pedigree errors is not new [1-3]. In this exercise we detect and describe the pedigree and genotyping errors in the GAW13 Framingham Heart Study data.

## Methods

### Pedigree error detection and correction

The program PREST [4] was used to detect pedigree errors. PREST estimates the probabilities, *p*_0_, *p*_1_, and *p*_2 _of two individuals sharing 0, 1, and 2 alleles identically by descent (IBD), respectively. We calculated this over all of the relationship pairs known to PREST (parent-offspring, full-sibs, half-sibs, avuncular, first-cousins, grandparent-grandchild, half-avuncular, half-first cousin, half-sib plus first-cousin, monozygotic twins, and unrelated) within and between pedigrees. Pedigree errors were first screened with PREST's analytical tests: conditional estimated identity by descent (EIBD), adjusted identity by state (AIBS), and IBS, in that order and where applicable, at α = 0.0001, to focus on the more significant problems. This index pair and their relatives were then examined more thoroughly using PREST's accompanying program ALTERTEST that can test two individuals for each of the 11 relationship classes.

PREST comes with an R script written by Dan Weeks to plot the IBD estimation of a single relative pair on a relationship triangle [5]. We modified this program to provide a scatter diagram of IBDs on the triangle. The result is an informative graphical summary of the pedigree errors in the sample. Pedigrees were drawn with PEDIGREE/DRAW [6].

### Genotyping error detection and correction

Genotyping errors are detected using SIMWALK [7,8]. SIMWALK2 applies a Markov-chain Monte Carlo method to data from the pedigree, population allele frequencies, and a genetic map to assign probabilities of mistyping for each genotype. Because this is a computationally intensive exercise, we examined genotyping errors only on chromosome 7.

We ran SIMWALK2 in two phases. In the first phase, Mendelian errors were detected and corrected independently for each marker. Marker genotypes were blanked (changed to a missing value) for all probabilities of mistyping above a given threshold. The threshold was chosen conservatively, i.e., to blank no more genotypes for a marker than necessary to calculate a likelihood for that marker. The mistyping probability was decremented from 1.0 until a calculable likelihood was reached.

In the second phase, genotypes that suggest improbable double recombination events were blanked. Mistyping probabilities were assigned using the genetic maps supplied with the GAW13 data. In this phase, the proportion of genotypes potentially blanked at a series of thresholds is plotted to provide a visual guide for choosing a threshold.

Following the corrections of the pedigree and genotype data, we recalculated for comparison, the chromosome 7 genetic map using MULTIMAP/CRIMAP [9,10].

## Results

### Pedigree errors

**Figure 1:**
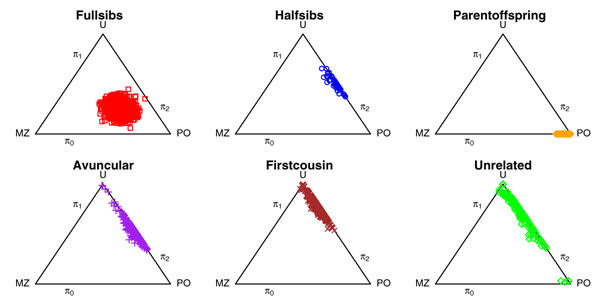
**Scatter diagrams of estimated IBDs on the relationship triangles for six relationship categories **Scatter diagrams of the IBD probabilities estimated by PREST on a relationship triangle reveal the extent of pedigree errors within a given relationship category. By far, the largest number of errors involves "unrelated" pairs that in fact appear to be related.

Scatter diagrams of the estimated IBD probabilities *p*_0_, *p*_1_, and *p*_2 _on relationship triangles are shown in Figure 1. These reveal almost no full-sibling errors. A few avuncular and first-cousin relationships are likely to be unrelated, and a great many unrelated individuals are likely in fact to be related, some being parent-offspring pairs.

**Figure 2:**
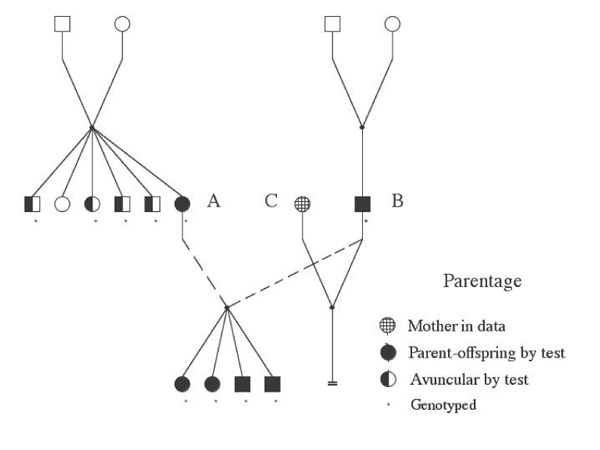
**Pedigree error example 1 **In the Framingham data the four offspring in the third generation were assigned mother C, but the tests identified A as the biological mother. B was confirmed as the biological father. The dashed lines indicate the corrected relationships.

Consistent observations among relatives were required before changes were made to the pedigrees. For example, the four unrelated pairs that are undoubtedly parent-offspring display the required parent-offspring relationships with a female in a disconnected pedigree, as determined by ALTERTEST and illustrated in Figure 2. All four were unrelated to the originally designated mother, now the stepmother. None of the other alternative tests suggested any other arrangement.

**Figure 3:**
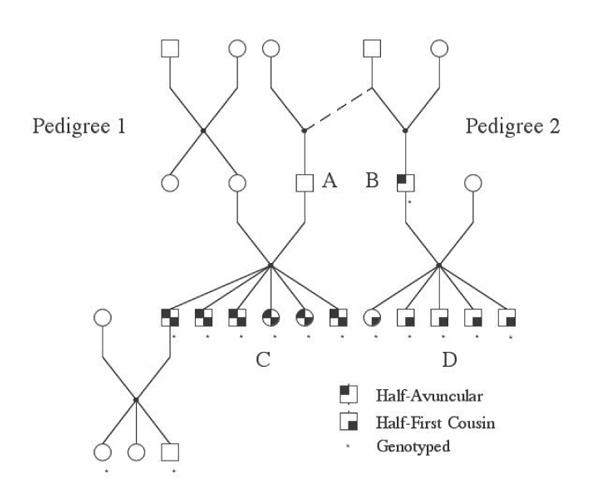
**Pedigree error example 2 **Pedigrees 1 and 2 were unrelated in the data. B was found to be a half-uncle to the siblings in C, and they in turn to be half-first-cousins to the siblings in D, supporting the joining of the two pedigrees as indicated with the dashed line.

In another example shown in Figure 3, two pedigrees were joined through two ungenotyped half-sibs, detected through consistent half-avuncular and half-first cousin relationships.

These simple examples belie the difficulties of correcting pedigree errors. In the Framingham data we were able to resolve the errors in 16 of 21 pedigrees, out of 329 pedigrees in total. The five unresolved pedigrees displayed patterns of errors that contradicted all testable alternative hypotheses, and were left unchanged.

### Genotyping errors

**Figure 4:**
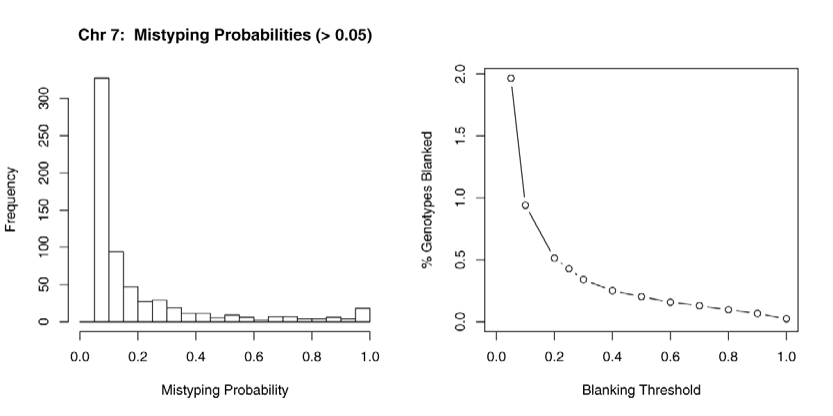
**Distribution of double-recombinant genotyping errors **The distribution of mistyping probabilities for the markers in chromosome 7 is shown on the left, and the proportion of genotypes blanked on the right, as determined by the mistyping probability thresholds on the x-axis. We chose to blank genotypes with mistyping probabilities greater or equal to 0.25.

Five Mendelian errors were revealed following the pedigree changes. The distribution of mistyping probabilities for the double-recombinant detection is shown in Figure 4. We chose a threshold probability of 0.25 to define erroneous genotypes, so as to blank without a too great loss of data. With this threshold, 142 genotypes were blanked.

Following the blanking, a new genetic map for the 21 markers on chromosome 7 was estimated using MULTIMAP/CRIMAP [9,10]. The new map was 167 cM in length, or 24 cM shorter than the map provided with the data. In comparison, the length of the corresponding Marshfield map is 175 cM [11].

## Discussion

The Framingham data were relatively free of pedigree errors, particularly those involving close relatives. The most frequent type of error involved individuals in the upper generations, detected through their descendants due to the lack of genotype data for the ancestors. These errors were frequently corrected through the joining of disconnected pedigrees. It is difficult to generalize these findings to other populations in which the types and distribution of pedigree errors may be quite different. Presumably in Framingham, kinship terms are usually given a biological interpretation. In other populations where this is not strictly the case, serious errors would seem possible.

The small number of Mendelian genotyping errors in the Framingham data that had not been detected previously became evident only following the pedigree corrections. Genotype errors that imply unlikely double recombination events were more common, the exact number depending on a definition of probable mistyping.
